# Development and validation of a nomogram for predicting clinically significant prostate cancer using serologic indices, multiparametric magnetic resonance imaging, and sound touch elastography parameters: a retrospective study

**DOI:** 10.3389/fonc.2025.1611706

**Published:** 2025-12-04

**Authors:** Kai Shu, Qian Chen, Junliang Chen, Qiang Xu, Yonghong Jin

**Affiliations:** 1 Department of Ultrasonography, Anqing Municipal Hospital, Anqing, Anhui, China; 2 Graduate School, Wannan Medical College, Wuhu, Anhui, China

**Keywords:** multiparametric magnetic resonance imaging, sound touch elastography, csPCa, cognitive fusion navigation-guided transperineal prostate biopsy, nomogram

## Abstract

**Objective:**

This study aimed to investigate independent risk factors for clinically significant prostate cancer (csPCa) using serologic indices, multiparametric magnetic resonance imaging (mpMRI), and sound touch elastography (STE), and to develop and validate a nomogram-based prediction model using the optimal model derived from these factors.

**Methods:**

A total of 240 patients who underwent ultrasound-guided transperineal prostate biopsy at Anqing Municipal Hospital between January 2024 and December 2024 were retrospectively enrolled. After applying exclusion criteria, 160 patients were included in the modeling cohort, which was divided into clinically significant prostate cancer (csPCa) and non-clinically significant prostate cancer (non-csPCa) groups based on pathological results. Additionally, 40 eligible patients from December 2024 to February 2025 were selected as the external validation cohort. Baseline data of the modeling cohort were collected, and independent risk factors for csPCa were identified using univariate and multivariate logistic regression analyses. The optimal model was selected by comparing with single-modal models, followed by developing a Nomogram prediction model. R language was used to plot decision curve analysis (DCA) for clinical utility evaluation, while receiver operating characteristic (ROC) curve and calibration curve were employed to assess predictive performance.

**Results:**

Multivariate logistic regression analysis identified The Prostate Imaging Reporting and Data System score, age, free-to-total (f/t) prostate-specific antigen (PSA), Emax, TZ-ratio (transition zone ratio), and lesion density as independent risk factors for csPCa (all *P* < 0.05). The combined independent risk factor model demonstrated superior predictive performance compared to single-modal models, with an area under the receiver operating characteristic curve (AUC) of 0.926, sensitivity of 88.0%, and specificity of 83.1%. A nomogram model was developed based on this optimal model. Decision curve analysis (DCA) revealed substantial clinical benefit and high usability across a wide range of threshold probabilities. Calibration curve validation showed excellent predictive accuracy, with close agreement between predicted and observed probabilities. Both internal and external validation cohorts confirmed consistent predictive performance of the model.

**Conclusion:**

The nomogram model integrating serologic indices, multiparametric mpMRI, and STE provides a more accurate and reliable tool for diagnosing csPCa, demonstrating substantial potential for clinical translation.

## Introduction

Prostate cancer (PCa), the most common malignant tumor of the male genitourinary system, has become the fifth leading cause of cancer-related deaths in men worldwide, accounting for 15% of the incidence of male malignant tumors (1). Early diagnosis and stratified management of PCa are essential to improve patient prognosis, in which serum prostate-specific antigen (PSA) test has been a core biomarker for PCa screening and diagnosis since its first clinical application in 1986, which can detect early PCa and reduce the risk of PCa (2, 3).

When the traditional screening threshold (PSA>4 ng/mL) is used, the specificity of PSA detection is 94% but only about 1/5 of men with elevated PSA are ultimately diagnosed with PCa (4). It is noteworthy that the majority of biopsy-positive patients with a PSA level in the gray zone (4–10 ng/mL) have a pathological diagnosis of a Gleason score of ≤6 clinically limited tumors that are clinically insignificant prostate cancers (5, 6). So it is not reasonable to make clinical decisions based on PSA alone, a strategy that will frequently detect clinically insignificant PCa and miss clinically significant prostate cancers (csPCa, Gleason score ≥7) (7).

How to achieve early and accurate identification of csPCa while effectively avoiding overdiagnosis of low-risk prostate cancer remains a key scientific challenge in the field of diagnosis and treatment. The traditional PSA-based screening model has shown significant limitations, prompting the academic community to actively explore new clinical markers and innovative technologies to improve the diagnostic efficacy of high-grade prostate cancer. With the continuous development of imaging, multiparametric magnetic resonance imaging (mpMRI) and ultrasound elastography have become the key technical tools for the localization and diagnosis of csPCa (8, 9). The Prostate Imaging-Reporting and Data System (PI-RADS) provides a standardized reading and diagnostic process for MRI images, which significantly improves the consistency of lesion detection (10). Ultrasound elastography provides effective detection and diagnostic results of glandular stiffness. glandular hardness can be effectively detected and quantified (11). Sound Touch Elastography (STE) represents an advanced ultrasound imaging technique rooted in two-dimensional shear wave elastography (2D-SWE), enabling real-time quantification of tissue hardness within targeted anatomical regions (12, 13). The clinical application of all these techniques has significantly improved the detection rate of csPCa, but it is of concern that there is still a lack of clinical studies that systematically integrate these multidimensional metrics, which may limit the construction of a system for the early and accurate diagnosis of csPCa.

The aim of this study was to integrate multidimensional indicators to explore the independent risk factors of csPCa, select the best model to construct and validate the Nomogram model, and evaluate the diagnostic efficacy of the model, through which individualized risk stratification was performed, while balancing the burden of biopsy and the risk of missed disease progression, to provide an innovative and quantitative tool for precise clinical decision-making.

## Materials and methods

### Study subjects

**Figure 1:**
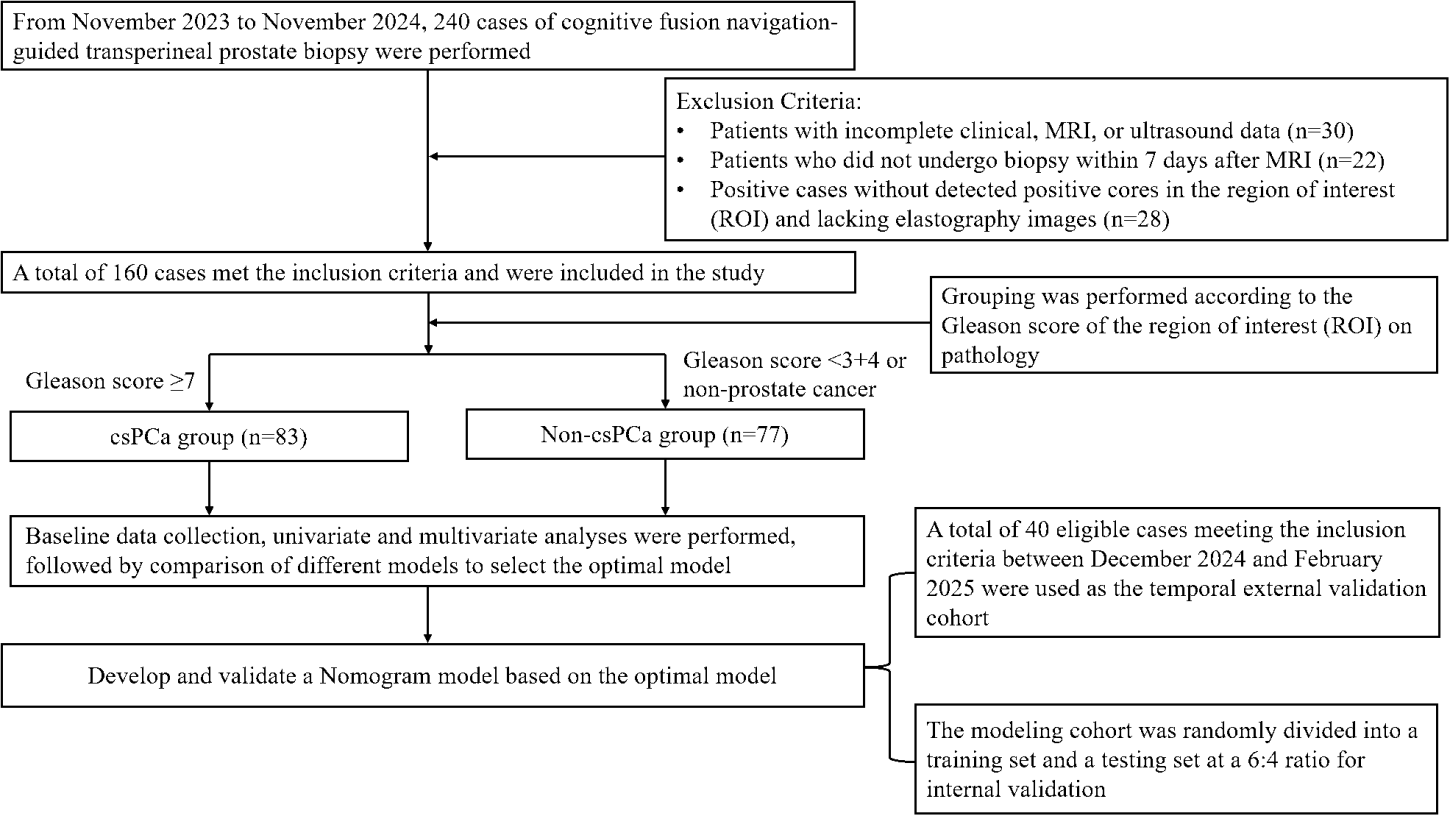
The flowchart of the study.

This is a single - center cross - sectional retrospective study. The study included patients who underwent an ultrasound-guided transperineal prostate biopsy at Anqing Municipal Hospital between January 2024 and February 2025. All clinicopathological data were collected from the hospital information system. Following the application of exclusion criteria, 160 patients were enrolled into the modeling group from 240 individuals between January 2024 and December 2024, while 40 patients were selected for the external validation group from 60 cases between December 2024 and February 2025. Based on pathological outcomes in this modeling group, there were 83 cases (51.9%) in the csPCa group and 77 cases (48.1%) in the non - csPCa group, in the external validation group, there were 22 cases (55%) in the CsPCa group and 18 cases (45%) in the non - csPCa group. The exclusion criteria of the study were as follows: patients with incomplete clinical, MRI, or ultrasound data; patients who did not undergo biopsy within 7 days after MRI; positive cases without detected positive cores in the region of interest (ROI) and lacking elastography images. This research was approved by the Medical Ethics Committee of Anqing Municipal Hospital. This study was conducted following the guiding principles of the Declaration of Helsinki, and all data were collected in compliance with the Health Insurance Portability and Accountability Act (HIPAA). As a retrospective study, it adheres to the 1975 Declaration of Helsinki, and a waiver of informed consent was obtained. The flowchart of the study can be seen in **Figure 1**.

### Research methods

Clinical baseline data collection: Relevant clinical baseline data of patients before puncture were retrospectively collected from the hospital’s electronic medical record system, such as age, free prostate-specific antigen (fPSA), total prostate-specific antigen (tPSA), and the ratio of free prostate-specific antigen to total prostate-specific antigen (f/tPSA), and the patients who were included in the study underwent serology for tumor markers of males after hospital admission. examination and complete magnetic resonance examination to ensure the timeliness and accuracy of the data. Total PSA and free PSA were measured using the Cobas e602 fully automated immunoassay analyzer (Roche, Switzerland).

MRI data were obtained using a 3.0 T Skyra ultra-high field magnetic resonance scanner (Siemens, Germany) with an 18-channel body matrix coil. Patients partially emptied their bladder before scanning, which began 1 cm superior to the pubic symphysis, covering at least the prostate and seminal vesicles. Imaging included T2-weighted imaging (T2WI) sequence (including the cross section, sagittal plane, and coronal plane), diffusion-weighted imaging (DWI) generated by single-shot spin echo planar imaging (SS-EPI) sequence, and dynamic contrast-enhanced MRI (DCE-MRI) was generated by fat-suppressed T1-weighted imaging (T1WI) sequence with 3D spoiled gradient echo, after rapid intravenous injection with gadopentetate dimeglumine (0.2 mmol/kg). Suspicious lesions were identified and marked by specialized radiologists, who recorded the maximum diameter of the lesions and assigned scores according to the Prostate Imaging-Reporting and Data System (PI-RADS) version 2.1. Additionally, glandular zone delimitation was performed, and maximum dimensions in three orthogonal planes were measured.

Prostate biopsy was performed as soon as possible after MRI scanning (all patients underwent biopsy within one week of MRI). Equipment used: Mindray Nuewa R9s color Doppler ultrasound diagnostic system with shear wave elastography (STE) technology, measurement analysis software, and ELC13-4U endocavity biplane probe. Curved array probe: Frequency 3.5–9.5 MHz, scanning angle 217°. Linear array probe: Frequency 3.2–12.8 MHz, scanning angle 40°.

The patient was placed in the lithotomy position and underwent routine disinfection. A bi - planar probe was inserted through the anus to conduct a transrectal ultrasound (TRUS) examination. The ultrasound image manifestations of the prostate in the horizontal and sagittal planes were observed. Attention was paid to the location and characteristics of abnormal echoes. Manual cognitive fusion was carried out in combination with MRI images to preliminarily determine the location of the lesion. If multiple suspicious lesions were detected by mpMRI, the lesion with the highest PI - RADS score was considered. The STE program was then initiated. The initial range of the probe was set to 0–70 kPa. The probe was kept gently touching the rectal wall as much as possible, and the sampling frame was adjusted to the maximum to include the left and right lobes of the prostate as much as possible. The region of interest was located according to the hardness indication and MRI images. After the color filling in the region of interest was satisfactory, the image was frozen. Calcified areas were avoided. The region of interest was selected using the tracing method, and the average Young’s modulus value (Emean) and the maximum Young’s modulus value (Emax) of the region of interest were measured. The measurement was repeated three times in the same region, and the average of the three measurements was taken as the final result. Subsequently, under the guidance of transrectal bi - planar ultrasound, a 12 - needle systematic prostate biopsy was performed. One to two additional targeted biopsies were taken from the region of interest. The puncture specimens from each needle were fixed and sent for biopsy. The histopathological analysis report of each sample included positive cores and the corresponding Gleason grades. The cases were grouped according to the pathological results of the puncture in the region of interest. In some cases, no positive cores were detected in the region of interest, but positive cores were found in other samples. These cases were not included in this study due to the lack of elastography data. Clinically significant prostate cancer (csPCa) was defined as a Gleason score of ≥3 + 4 = 7. All the above operations were performed by physicians at or above the intermediate level in this specialty.

### Parameter calculation

The prostate volume (PV) and the transition zone volume (TZV) are calculated using the ellipsoid formula (volume = length × width × height × π/6, in cubic centimeters). The peripheral zone volume (PZV) is calculated as the difference between the PV and the TZV. The lesion density is calculated as the ratio of the longest diameter of the lesion (in millimeters) to the prostate volume (in cubic centimeters). The TZ-ratio is defined as the ratio of the transition zone volume to the prostate volume.

### Statistical methods

All statistical analyses were performed using Excel 2019 (Microsoft Corporation, Redmond, WA, USA), IBM SPSS Statistics 26.0 (IBM Corp, Armonk, NY, USA), and R 4.4.0 (R Foundation for Statistical Computing, Vienna, Austria). Continuous variables with normal distribution were expressed as mean ± standard deviation (mean [SD]), while non-normally distributed variables were presented as medians with 25th and 75th percentiles. Categorical variables were reported as counts and percentages (%). Baseline characteristics were compared across groups using χ² tests or Fisher’s exact tests for categorical variables. For normally distributed continuous variables, one-way analysis of variance (ANOVA) was applied; non-normally distributed variables were analyzed using the Kruskal-Wallis H test or Mann-Whitney U test. Spearman correlation coefficients were calculated to assess relationships between variables. Univariate and multivariate logistic regression models were constructed to identify independent predictors of csPCA Covariates with significant associations in univariate analysis (p < 0.05) were included in the multivariate model. Forest plots were used to visualize adjusted odds ratios (ORs) and 95% confidence intervals (CIs). A predictive nomogram was developed based on significant variables from multivariate analysis. Discrimination was evaluated using receiver operating characteristic (ROC) curves, with the area under the curve (AUC) reported. Calibration was assessed via Hosmer-Lemeshow goodness-of-fit testing (p > 0.05 indicating adequate fit), and clinical utility was validated through decision curve analysis (DCA). The Delong test was used to compare ROC curves and evaluate differences in the AUC among various models. All statistical tests were two-tailed, with p < 0.05 considered statistically significant.

## Result

### Comparison of clinical baseline data between csPCa group and non-csPCa group

A total of 160 subjects were included in the modeling group of this study. Clinical baseline data of the patients were retrospectively collected (**Table 1**). According to the pathological results, the csPCa group (n=83, 51.9%) had a median age of 74.00 (68.00, 77.00), and the non-csPCa group (n=77, 48.1%) had a median age of 67.00 (62.00, 73.00). In the comparison of baseline characteristics, several variables showed significant differences, including age; tPSA; fPSA; f/t PSA; Emax; Emean; PV; TZV; TZ-ratio; Lesion Density; PI-RADS score (P<0.05). Compared with the non-csPCa group, the csPCa group had a higher median age (74.00 years vs. 67.00 years), higher levels of tPSA and fPSA, but a lower f/t PSA. Among elastography-related indicators, Emax and Emean in the csPCa group were significantly higher than those in the non-csPCa group. PV, TZV, and TZ-ratio were higher in the non-csPCa group. In addition, the csPCa group had a higher lesion density and a relatively higher PI-RADS score, and there was also a significant difference in PZV between the two groups (P = 0.032).

**Table 1:** Comparison of clinical baseline data between csPCa group and non-csPCa group.

Factors	All	csPCa(n=83)	Non- csPCa(n=77)	Z/χ²	P
Age (years)	70.50(63.00,76.00)	74.00(68.00,77.00)	67.00(62.00,73.00)	-4.094	<0.001
tPSA (ng/ml)	12.85(9.51,21.37)	16.34(11.95,45.75)	11.38(8.56,14.46)	-4.646	<0.001
fPSA (ng/ml)	2.30(1.42,4.03)	2.68(1.59,7.59)	2.20(1.17,3.06)	-2.795	0.005
fPSA/tPSA	0.16(0.10,0.25)	0.14(0.09,0.21)	0.19(0.12,0.26)	-2.853	0.004
Emax(kPa)	73.04(52.25,89.30)	78.54(59.23,101.36)	59.83(48.58,77.44)	-4.692	<0.001
Emean(kPa)	43.31(36.64,59.30)	55.33(39.58,67.58)	39.41(32.19,52.85)	-4.791	<0.001
PV(cm³)	49.07(32.37,70.75)	41.50(26.75,55.26)	56.28(42.13,95.46)	-4.043	<0.001
TZV(cm³)	29.85(18.07,44.44)	23.12(14.91,32.87)	35.38(25.16,59.75)	-4.687	<0.001
PZV(cm³)	18.61(12.54,29.21)	17.48(11.71,24.62)	20.81(13.70,35.25)	-2.140	0.032
TZ-ratio	0.62(0.54,0.68)	0.59(0.51,0.63)	0.67(0.60,0.70)	-4.777	<0.001
Lesion Density(mm/cm³)	0.35(0.21,0.54)	0.46(0.31,0.64)	0.28(0.17,0.40)	-5.120	<0.001
PI-RADS score				50.721	<0.001
2	5(3.1%)	1(1.2%)	4(5.2%)		
3	67(41.9%)	15(18.1%)	52(67.5%)		
4	46(28.7%)	30(36.1%)	16(20.8%)		
5	42(26.3%)	37(44.6%)	5(6.5%)		

Data are expressed as n (%), mean ± SD, or median (interquartile range). tPSA=total prostate-specific antigen; fPSA=free prostate-specific antigen; fPSA/tPSA=free-to-total (f/t) prostate-specific antigen; Emax=the maximum value of Young’s modulus in the region of interest; Emean=the minimum value of Young’s modulus in the region of interest; PV= prostate volume; TZV=transition zone volume; PZV=peripheral zone volume; PI-RADS score=density prostate imaging-reporting and data system score. The t-value is a statistical value used to evaluate the difference between a sample and the population; the χ²-value represents the degree of deviation between the observed values and the theoretical values; the P-value reflects the degree of confidence in rejecting the null hypothesis, and a P-value less than 0.05 indicates that the difference is statistically significant.

### Variable assignment in logistic regression analysis and independent diagnostic factors for csPCa

**Figure 2:**
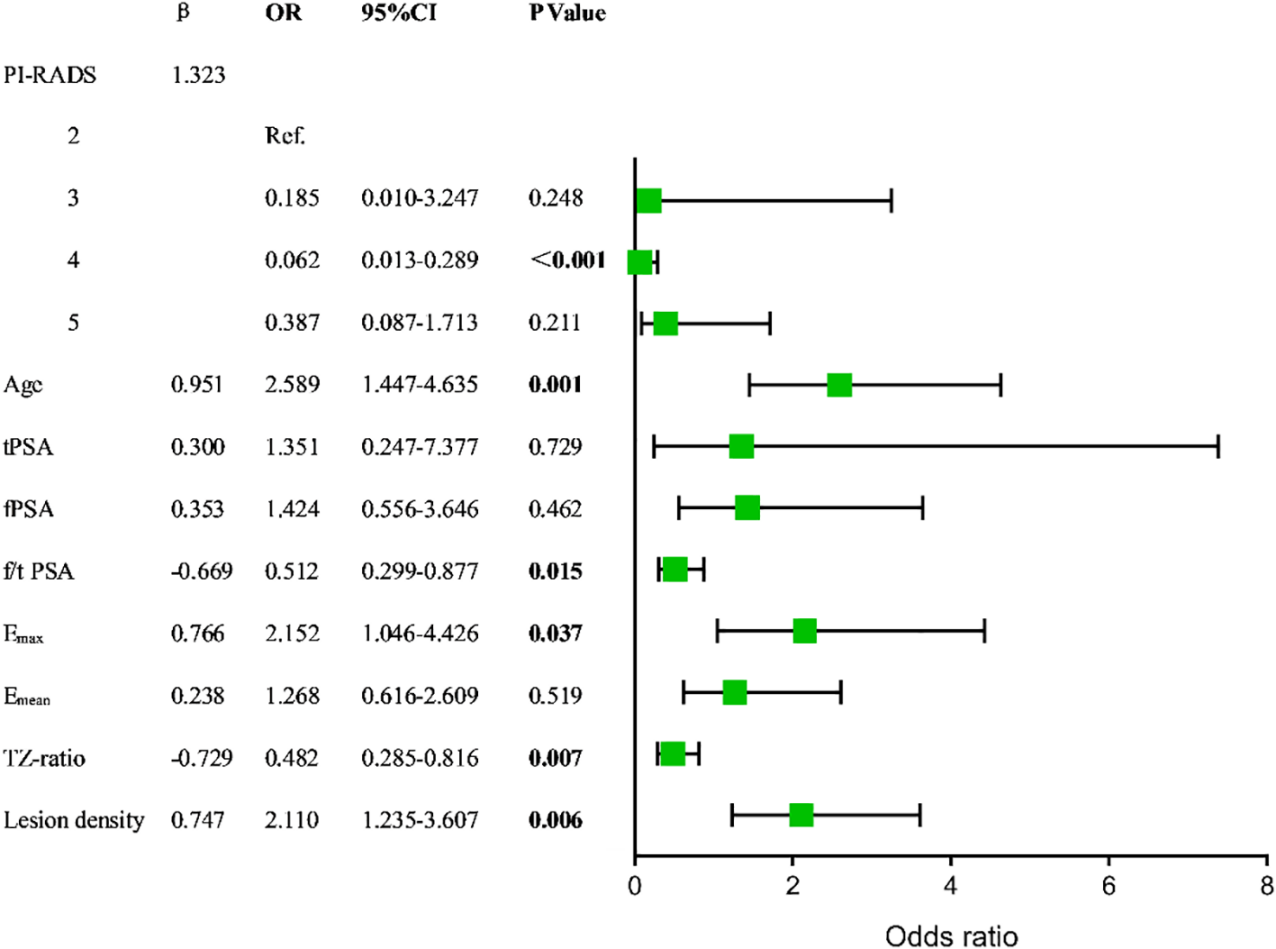
Forest plot.

The TZ-ratio is a measure of the volume of the transition zone between the prostate and the PV, while the peripheral zone volume is calculated by taking TZV out of PV. Both PV and TZV were excluded from the univariate analysis due to their linear relationship and standard deviations. In the logistic regression analysis, variables were assigned specific cut-off values. (**Table 2**, **Supplementary Table 1**). The model identified several independent diagnostic factors for csPCa, including age, f/t PSA, Emax, TZ-ratio, lesion density, and PI-RADS score. These coefficients indicate their impact on the probability of diagnosing csPCa (**Supplementary Table 2**, **Figure 2**). Collinearity diagnosis using Variance Inflation Factor (VIF) demonstrated no significant multicollinearity among the six independent factors, with VIF values of 1.2, 1.1, 1.1, 1.1, 1.1, and 1.1 respectively (all <10) (**Supplementary Table 3**).

**Table 2:** Assignment of variables in logistic regression analysis.

Variable	Variable type	Assignment details
Age	(X)	≥69 years =1, <69 years =0
tPSA	(X)	≥14.898 = 1, <14.898 = 0
fPSA	(X)	≥5.553 = 1, <5.553 = 0
f/t PSA	(X)	<0.205 = 1, ≥0.205 = 0
Emean	(X)	≥57.355 = 1, <57.355 = 0
Emax	(X)	≥72.535 = 1, <72.535 = 0
TZ ratio	(X)	<0.63 = 1, ≥0.63 = 0
Lesion density	(X)	≥0.405 = 1, <0.405 = 0
PI-RADS score	(X)	≥3.5 = 1, <3.5 = 0
Disease Type	(Y)	csPCa =1, Non-csPCa =0

### Logistic regression equation and the establishment of nomogram

**Figure 3:**
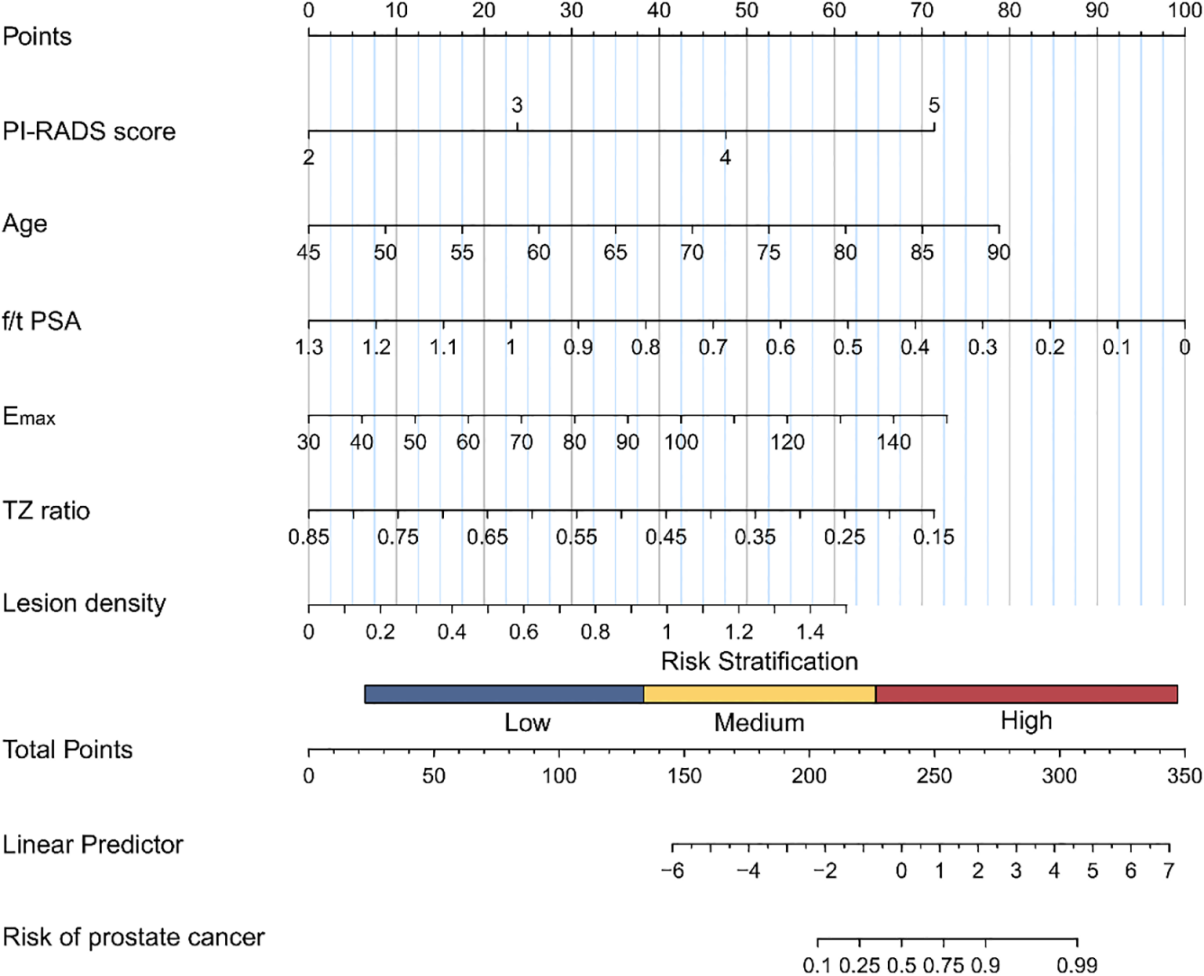
Nomogram model for csPCa. PI-RADS score=density prostate imaging-reporting and data system score. f/t PSA=free-to-total (f/t) prostate-specific antigen; Emax=the maximum value of Young's modulus in the region of interest; TZ ratio=the ratio of the transition zone volume to the prostate volume; lesion density=the ratio of the longest diameter of the lesion to the prostate volume.

The logistic regression equation is as follows: Logit (p)= -4.723 + 0.951× Age – 0.669 × f/tPSA + 0.766 × Emax – 0.729 × TZ ratio + 0.747 × Lesion density + 1.323× PI-RADS score. Nomograms were constructed using R software and the rms package to visualize the model, as shown in **Figure 3**. From the figure, one can obtain the corresponding score for each indicator, and the predicted probability corresponding to the total points represents the probability of being diagnosed with csPCa.

### Analysis of ROC curves for different models

**Figure 4:**
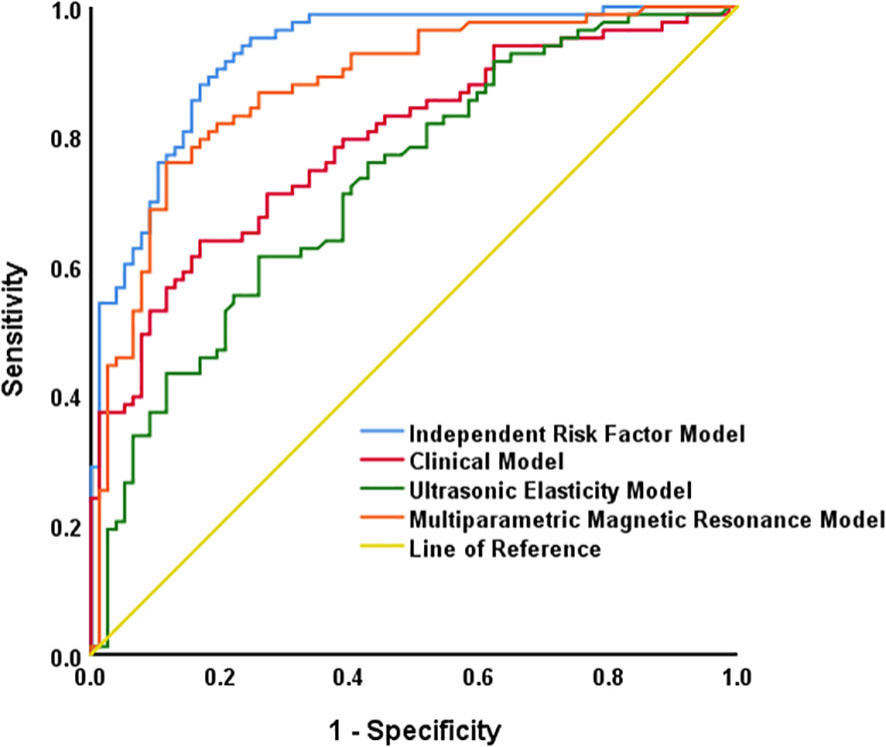
ROC curves of different diagnostic models. PI-RADS score=density prostate imaging-reporting and data system score. f/t PSA=free-to-total (f/t) prostate-specific antigen; Emax=the maximum value of Young's modulus in the region of interest; TZ ratio=the ratio of the transition zone volume to the prostate volume; lesion density=the ratio of the longest diameter of the lesion to the prostate volume. Total points: Sum of the scores of all predictive indicators.

Based on the sources of baseline factors with statistical significance in univariate analysis, other predictive models were constructed, namely Model 1 (Clinical Model): Age + tPSA +fPSA + f/t PSA; Model 2 (Ultrasonic Elasticity Model): Emax+ Emean; Model 3 (Magnetic Resonance Model): TZ-ratio + lesion density + PI-RADS score; and Model 4 (Independent Risk Factor Model): PI-RADS score + Age + f/t PSA + Emax+ TZ-ratio + lesion density. Compared with other models, the independent risk factor model showed a higher AUC value, indicating better diagnostic accuracy, and all differences were statistically significant (all P<0.05), (**Table 3**, **Table 4**; **Figure 4**).

**Table 3:** ROC parameters of different diagnostic models.

Model	AUC	Youden-index	Cut-off	Sensitivity	Specificity	95%CI
Model 1	0.790	0.470	0.560	0.639	0.831	0.722-0.859
Model 2	0.727	0.345	0.545	0.614	0.740	0.650-0.805
Model 3	0.875	0.642	0.584	0.759	0.883	0.821-0.930
Model 4	0.926	0.711	0.506	0.880	0.831	0.886-0.965

Model 1: Clinical Model; Model 2: Ultrasonic Elasticity Model; Model 3: Multiparametric Magnetic Resonance Model; Model 4: Independent Risk Factor Prediction Model

**Table 4:** Comparison of AUC between different diagnostic models.

Model comparison	AUC difference	SE	95%CI	Z	P
Model 1 vs Model 4	0.135	0.234	0.071-0.200	4.105	<0.001
Model 2 vs Model 4	0.198	0.244	0.119-0.277	4.922	<0.001
Model 3 vs Model 4	0.050	0.218	0.011-0.089	2.537	0.011

Model 1: Clinical Model; Model 2: Ultrasonic Elasticity Model; Model 3: Multiparametric Magnetic Resonance Model; Model 4: Independent Risk Factor Prediction Model

### Internal validation

The modeling cohort was randomly divided into a training set and a testing set at a 6:4 ratio are presented in **Supplementary Table 4**. No significant differences were observed between the two groups (P>0.05).For internal validation, the Hosmer-Lemeshow goodness-of-fit test showed excellent calibration in both the training set (χ²=5.418, P = 0.712) and the testing set (χ²=2.930, P = 0.939), with P>0.05 indicating no significant deviation from perfect calibration. Calibration curves demonstrated high consistency between predicted and observed probabilities (**Supplementary Figures 1A, B**). Decision Curve Analysis (DCA) revealed the Nomogram’s favorable clinical utility, with substantial net benefit across a wide range of threshold probabilities (**Supplementary Figures 2A, B**). Receiver Operating Characteristic (ROC) curve analysis yielded an AUC of 0.952 in the training set and 0.922 in the testing set (**Supplementary Figures 3A, B**). Delong’s test comparing the two ROC curves showed no significant difference (P = 0.07), confirming the model’s stable performance across datasets.

### External validation

Clinical baseline data in the external validation cohort are presented in **Supplementary Table 5**. The Hosmer-Lemeshow test results (χ²=7.779; P = 0.455) indicated high consistency between predicted and observed values via the calibration curve (P = 0.455>0.05), confirming the model’s robust predictive performance. The DCA revealed the Nomogram’s favorable clinical utility. The external validation cohort achieved an AUC of 0.924, demonstrating excellent discriminatory ability for csPCa (**Supplementary Figures 4A–C**).

## Discussion

Despite the availability of multiple screening methods, the diagnosis and treatment of csPCa remain a major public health challenge worldwide—particularly in Asia, where over half of all prostate cancer patients are diagnosed with csPCa (14, 15). Current prostate cancer screening primarily relies on PSA testing; however, PSA-detected cancers often represent indolent lesions that may never cause symptoms or require treatment. Such screening programs may lead to overdiagnosis and overtreatment of low-risk patients (16, 17). Our study supports the need to optimize existing screening strategies to improve csPCa detection. Although univariate analysis showed higher PSA levels in the csPCa group compared to the non-csPCa group, multivariate analysis identified PSA as a non-independent risk factor. While elevated PSA may correlate with csPCa risk, it is not a reliable diagnostic marker—a finding consistent with previous studies (18). These results underscore the urgent need to explore alternative predictive factors for csPCa.

Previous studies have confirmed that age is a key risk factor for csPCa, and with advancing age, the incidence rate approximately doubles every 14 years (19). In our study, older age was associated with higher csPCa risk, underscoring the importance of early intervention in elderly patients.

The f/t PSA is widely recognized as a critical parameter for distinguishing benign prostatic hyperplasia from prostate cancer in men with PSA levels between 4–10 ng/mL (20). Fangming Wang et al. reported that f/t PSA had superior diagnostic performance compared to total PSA in the PSA gray zone (AUC = 0.551 vs. 0.364) (21). However, our study did not directly compare the diagnostic efficiency of individual independent predictors.

Compared to normal lesions, the hardness of malignant lesions increased significantly (22). Therefore, monitoring and quantifying tissue stiffness are critical for detecting csPCa. Elastography, a rapidly evolving non-invasive diagnostic imaging technique, can visualize and quantify tissue hardness and elasticity in real-time (23). Scholar Abraham T Oladimeji utilized SWE for diagnosing high-grade prostate cancer, achieving favorable diagnostic performance (AUC: 0.935, specificity: 94.59%, sensitivity: 84.21%) (9).

In this study, STE – a modality enabling simultaneous display of 2D grayscale and elasticity images for lesion localization and measurement – was employed. STE has also demonstrated diagnostic efficacy in liver fibrosis (AUC: 0.706) (24). Unlike prior studies relying on elastography scoring systems, our research used Young’s modulus values to quantitatively assess suspicious lesions and performed cognitive fusion navigation-targeted biopsy with elasticity image guidance.

Two elasticity parameters were evaluated: Emax and Emean. Emax emerged as an independent risk factor, with higher values observed in positive cases. According to Ouyang Lu’s findings (12), Emax alone yielded an AUC of 0.748, specificity of 84.9%, sensitivity of 64.6%, and optimal cutoff value of 67.47. Combining Emax with prostate-specific antigen density (PSAD) improved diagnostic accuracy (AUC: 0.909).Notably, Emean did not exhibit significant predictive value in our study, differing from Ouyang Lu’s results. Given limited existing research on Emean, further investigations with larger sample sizes are warranted to explore its potential role in predicting csPCa.

In recent years, mpMRI has gradually become a recommended imaging modality for prostate cancer diagnosis. According to the European Association of Urology guidelines, performing an MRI before biopsy is always beneficial for patients. mpMRI demonstrates high sensitivity for csPCa, especially in cases with lesions larger than 10 mm (25). The introduction of the Prostate Imaging-Reporting and Data System (PI-RADS) scoring system has provided a standardized framework for interpreting MRI images and diagnostic workflows, enabling more accurate identification and diagnosis of csPCa (26). The PI-RADS system exhibits a high positive predictive value, which even increases with higher scores—for example, lesions with a PI-RADS score of 5 have a positive predictive value as high as 94% in some studies (27). A recent meta-analysis reported cancer detection rates of 16%, 59%, and 85% for PI-RADS 3, 4, and 5 lesions, respectively (28). In our study, the detection rates for PI-RADS 3, 4, and 5 lesions were 22.4%, 65%, and 88%, slightly higher than those of previous related research.

Both the TZ-ratio (29) and lesion density (18) are predictors derived from mpMRI studies. Since benign prostatic hyperplasia primarily originates in the transition zone, the proportion of transition zone volume in benign hyperplasia is higher. Previous ROC analysis showed that among several prostate volume-related variables, the TZ-ratio yielded the highest area under the curve (AUC, 0.746; 95% CI 0.636–0.856), indicating the best diagnostic efficacy. Additionally, lesion density is a strong predictor of csPCa in targeted biopsy (AUC: 0.743, sensitivity 75.1%, specificity 59.7%, performing well across subgroups of different PI-RADS scores. Lesion density emphasizes the relationship between lesion size and prostate volume, helping to improve the diagnostic effectiveness of targeted biopsy. As an indicator of lesion prominence within the prostate, larger lesions in smaller prostate volumes are more easily localized correctly, increasing the chance of successful biopsy. Moreover, increased lesion density may indicate a higher lesion burden within a smaller glandular area.

The abovementioned predictive factors have demonstrated good predictive efficacy in previous studies, but few studies have combined these factors for analysis. This cross-sectional retrospective study integrated risk factors to develop a risk prediction model. This study compared unimodal data models and found the independent risk factor model had better diagnostic performance, thus avoiding misdiagnosis from reliance on single markers. A Nomogram model was constructed, validated by Hosmer-Lemeshow test (P>0.05), calibration curves, and DCA, which confirmed its clinical utility. By combining tissue elasticity data from STE with mpMRI’s high-resolution imaging, the model comprehensively evaluates prostate lesions, enhancing high-risk tumor detection. Clinically, it optimizes diagnostic workflows by stratifying biopsy eligibility, reducing unnecessary procedures in low-risk patients while ensuring timely intervention for high-risk individuals. The model is cost-effective and non-invasive, making it feasible for widespread adoption in csPCa screening and personalized treatment strategies.

While our predictive model demonstrated favorable performance, several limitations should be acknowledged. Firstly, this was a single-center, cross-sectional retrospective study inherently prone to recall and selection biases. Multicenter cohort studies are warranted to validate the model’s generalizability. Second, the small sample size (all patients from Anqing Municipal Hospital) may limit statistical power. Future research should expand sample size and explore combinatorial testing strategies to enhance model precision. Third, pathological diagnosis was based on targeted prostate biopsy results, which may have false-negative rates. Long-term follow-up with PSA monitoring for negative patients is ongoing. In future studies, we plan to adjust selection strategies and include multi-regional, multi-center patient data to improve model robustness.

## Data Availability

The raw data supporting the conclusions of this article will be made available by the authors, without undue reservation.
