# Supplementary material for: Development and validation of a nomogram for predicting clinically significant prostate cancer using serologic indices, multiparametric magnetic resonance imaging, and sound touch elastography parameters: a retrospective study
Source: Front Oncol. 2025 Dec 4;15:1611706. doi: 10.3389/fonc.2025.1611706 (PMC12711473; doi:10.3389/fonc.2025.1611706)
Supplement: Supplementary file 1 [file DataSheet1.docx]

Supplementary Material

**Supplementary Figures and Tables**

Supplementary table 1. Assignment of variables in logistic regression analysis

|  | Cut-off | Specificity | Sensitivity | Youden-index | 95%CI |
| --- | --- | --- | --- | --- | --- |
| Age | 69 | 0.649 | 0.723 | 0.372 | 0.603-0.772 |
| tPSA | 14.898 | 0.779 | 0.578 | 0.357 | 0.632-0.791 |
| fPSA | 5.553 | 0.922 | 0.337 | 0.259 | 0.542-0.714 |
| f/t PSA | 0.205 | 0.583 | 0.557 | 0.140 | 0.498-0.641 |
| Emean | 57.355 | 0.870 | 0.470 | 0.340 | 0.636-0.794 |
| Emax | 72.535 | 0.662 | 0.663 | 0.325 | 0.641-0.798 |
| TZ ratio | 0.63 | 0.678 | 0.652 | 0..330 | 0.615-0.741 |
| Lesion density | 0.405 | 0.792 | 0.614 | 0.406 | 0.657-0.813 |
| PI-RADS score | 3.5 | 0.727 | 0.807 | 0.534 | 0.733-0.872 |
| Note: tPSA=total prostate-specific antigen; fPSA=free prostate-specific antigen; f/t PSA=free-to-total (f/t) prostate-specific antigen; Emax=the maximum value of Young's modulus in the region of interest; Emean=the minimum value of Young's modulus in the region of interest; PV= prostate volume; TZV=transition zone volume; PZV=peripheral zone volume; PI-RADS score=density prostate imaging-reporting and data system score. | | | | | |

Supplementary table 2. Univariate and multivariate regression analyses for csPCa

|  | Univariate Analysis | | |  | Multifactorial Analysis | | | |
| --- | --- | --- | --- | --- | --- | --- | --- | --- |
|  | 95%CI | OR | P |  | 95%CI | OR | P | β |
| Age | 1.316-2.639 | 1.863 | **<0.001** |  | 1.447-4.635 | 2.589 | **0.001** | 0.951 |
| tPSA | 3.004-34.992 | 10.253 | **0.001** |  | 0.247-7.377 | 1.351 | 0.729 | 0.300 |
| fPSA | 1.440-3.918 | 2.375 | **0.001** |  | 0.556-3.646 | 1.424 | 0.462 | 0.353 |
| f/t PSA | 0.383-0.899 | 0.587 | **0.014** |  | 0.299-0.877 | 0.512 | **0.015** | -0.669 |
| Emax | 1.588-3.384 | 2.318 | **<0.001** |  | 1.046-4.426 | 2.152 | **0.037** | 0.766 |
| Emean | 1.622-3.433 | 2.360 | **<0.001** |  | 0.616-2.609 | 1.268 | 0.519 | 0.238 |
| PZV | 0.962-1.006 | 0.984 | 0.157 |  |  |  |  |  |
| TZ-ratio | 0.340-0.734 | 0.499 | **<0.00**1 |  | 0.285-0.816 | 0.482 | **0.007** | -0.729 |
| Lesion density | 1.622-3.841 | 2.496 | **<0.001** |  | 1.235-3.607 | 2.110 | **0.006** | 0.747 |
| PI-RADS |  |  | **<0.001** |  |  |  | **0.001** | 1.323 |
| 2 | Ref. | | | | | | |  |
| 3 | 0.003-0.336 | 0.034 | **0.005** |  | 0.010-3.247 | 0.185 | 0.248 |  |
| 4 | 0.013-0.117 | 0.039 | **<0.001** |  | 0.013-0.289 | 0.062 | 0.001 |  |
| 5 | 0.083-0.772 | 0.253 | **0.016** |  | 0.087-1.713 | 0.387 | 0.211 |  |
| Note: tPSA=total prostate-specific antigen; fPSA=free prostate-specific antigen; f/t PSA=free-to-total (f/t) prostate-specific antigen; Emax=the maximum value of Young's modulus in the region of interest; Emean=the minimum value of Young's modulus in the region of interest; PV= prostate volume; TZV=transition zone volume; PZV=peripheral zone volume; PI-RADS score=density prostate imaging-reporting and data system score. | | | | | | | | |

Supplementary table 3. Collinearity Analysis of Independent Risk Factors

| Factors | Tolerance | VIF | t | P |
| --- | --- | --- | --- | --- |
| PI-RADS score | 0.853 | 1.172 | 6.072 | <0.001 |
| Age | 0.872 | 1.147 | 3.784 | <0.001 |
| f/t PSA | 0.933 | 1.072 | -3.296 | <0.001 |
| Emax | 0.950 | 1.053 | 4.021 | <0.001 |
| TZ-ratio | 0.917 | 1.090 | -3.142 | 0.002 |
| Lesion density | 0.893 | 1.119 | 3.070 | 0.003 |

Supplementary table 4. Comparison of clinical baseline data between training Set Group and testing set Group

|  | ALL  *N=160* | test  *N=64* | train  *N=96* | *P* |
| --- | --- | --- | --- | --- |
| Age (years) | 69.8 (8.66) | 69.9 (9.52) | 69.8 (8.08) | 0.937 |
| tPSA(ng/ml) | 25.4 (40.1) | 25.1 (50.2) | 25.6 (32.1) | 0.945 |
| fPSA(ng/ml) | 4.06 (4.62) | 4.26 (5.09) | 3.92 (4.29) | 0.665 |
| f/t PSA | 0.16 [0.10;0.24] | 0.16 [0.10;0.25] | 0.16 [0.11;0.24] | 0.845 |
| Emax( kPa) | 73.0 [52.3;89.2] | 70.8 [52.3;82.8] | 75.2 [52.3;90.2] | 0.382 |
| Emean( kPa) | 48.1 (16.5) | 47.4 (17.6) | 48.5 (15.9) | 0.698 |
| TZ ratio | 0.62 [0.54;0.68] | 0.62 [0.55;0.68] | 0.62 [0.54;0.67] | 0.751 |
| Lesion density(mm/cm³) | 0.35 [0.21;0.54] | 0.34 [0.20;0.52] | 0.38 [0.23;0.54] | 0.366 |
| PI RADS score |  |  |  | 0.452 |
| 2 | 5 (3.12%) | 3 (4.69%) | 2 (2.08%) |  |
| 3 | 67 (41.9%) | 29 (45.3%) | 38 (39.6%) |  |
| 4 | 46 (28.7%) | 19 (29.7%) | 27 (28.1%) |  |
| 5 | 42 (26.2%) | 13 (20.3%) | 29 (30.2%) |  |
| csPCa |  |  |  | 0.821 |
| No | 77 (48.1%) | 32 (50.0%) | 45 (46.9%) |  |
| Yes | 83 (51.9%) | 32 (50.0%) | 51 (53.1%) |  |
| Note: tPSA=total prostate-specific antigen; fPSA=free prostate-specific antigen; f/t PSA=free-to-total (f/t) prostate-specific antigen; Emax=the maximum value of Young's modulus in the region of interest; Emean=the minimum value of Young's modulus in the region of interest; PV= prostate volume; TZV=transition zone volume; PZV=peripheral zone volume; PI-RADS score=density prostate imaging-reporting and data system score. | | | | |


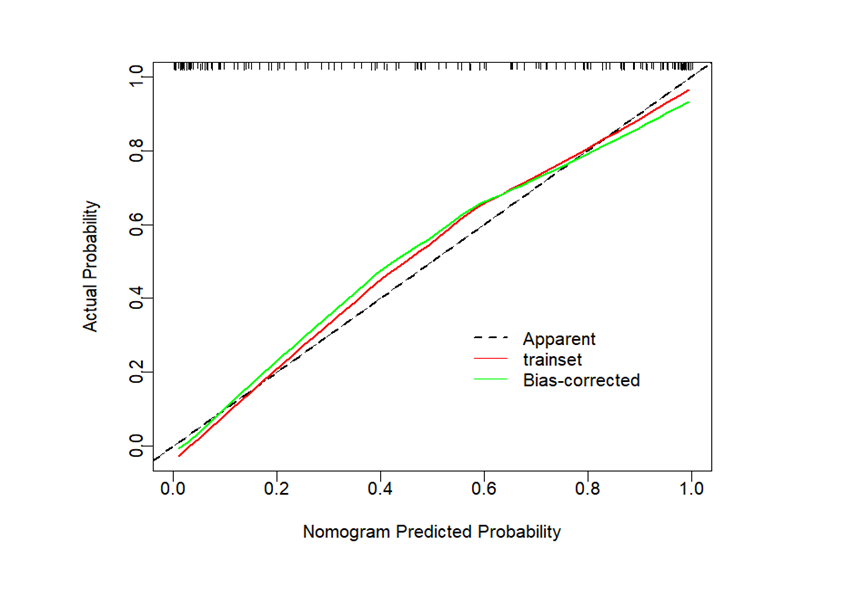


1a


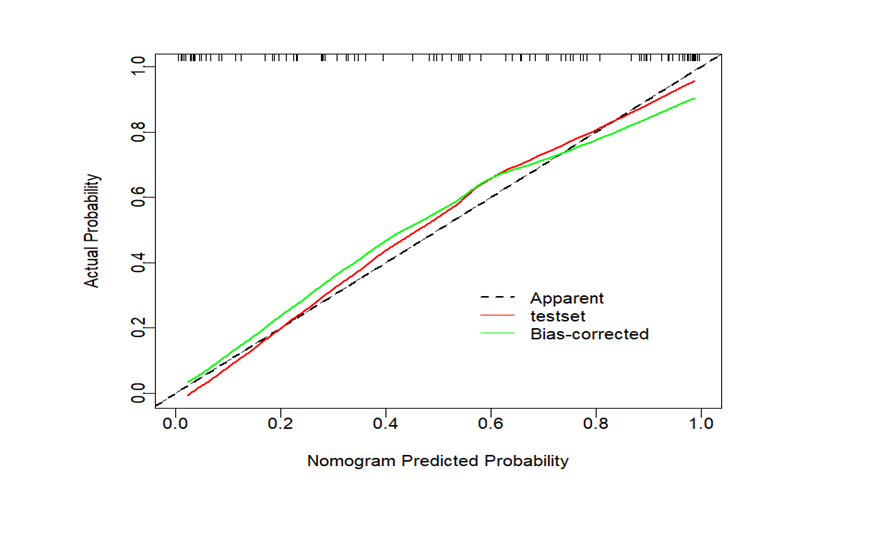


1b

Supplementary figures 1a and 1b. Calibration curves for the nomogram model in the training set and testing set.


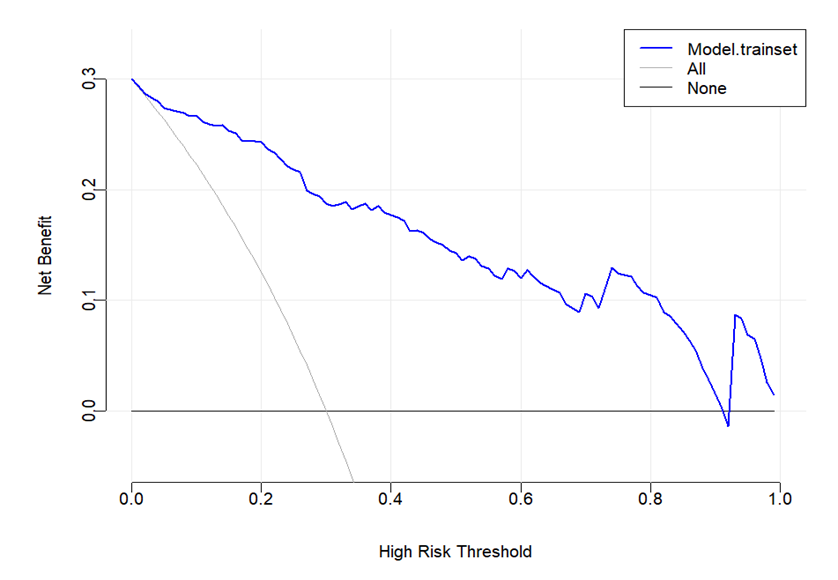


2a


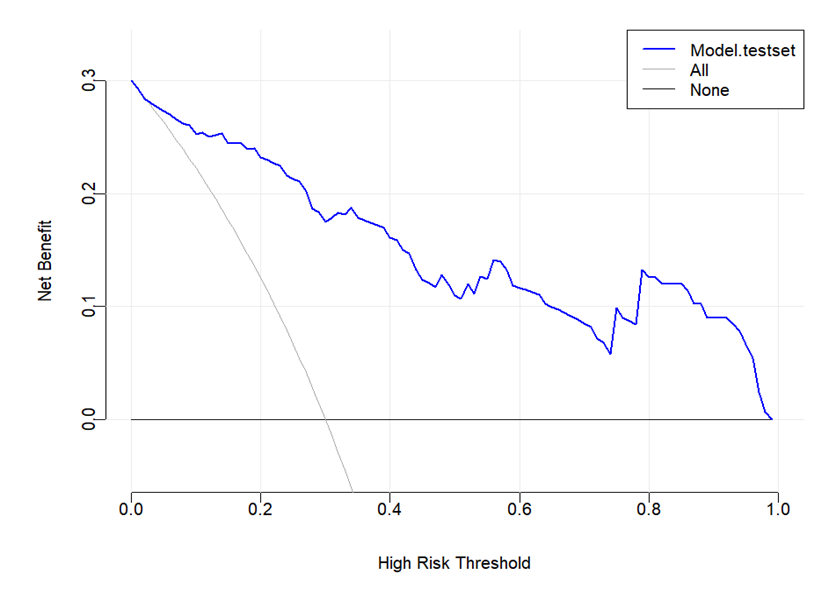


2b

Supplementary figures 2a and 2b. The decision curve analysis for the nomogram model in the training set and testing set.


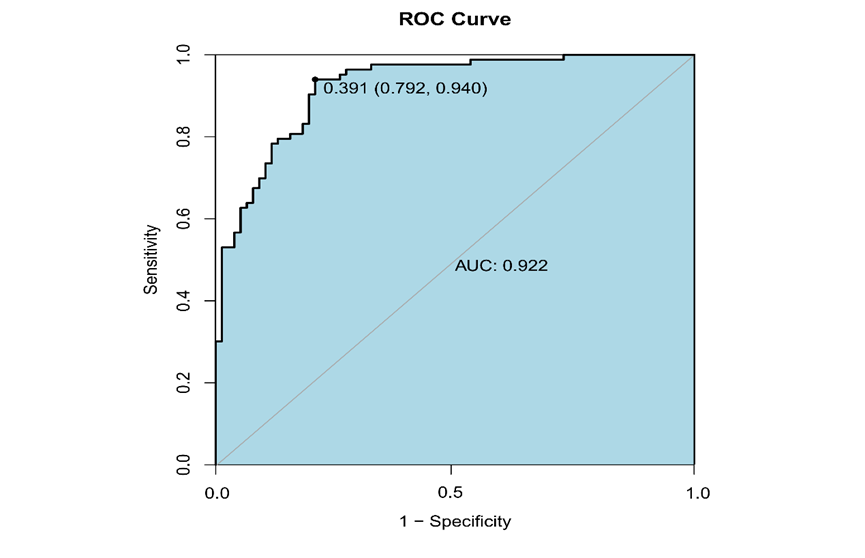


3a


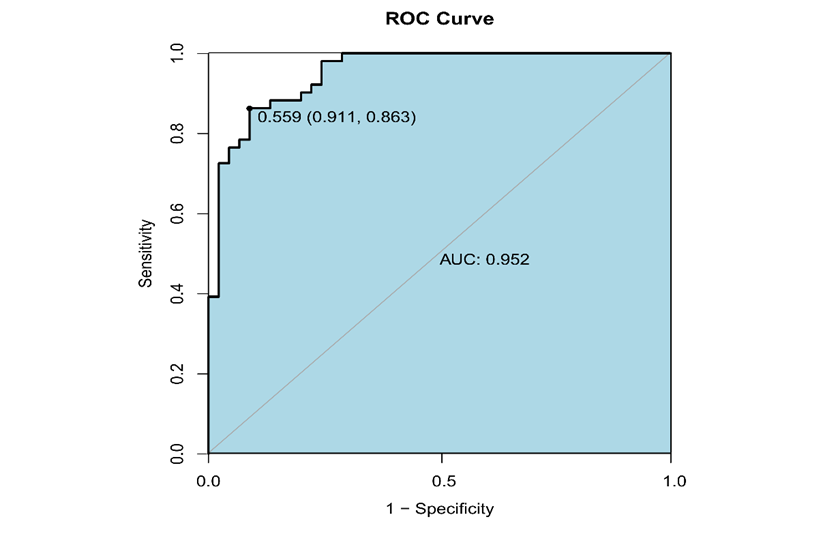


3b

Supplementary figures 3a and 3b. ROC in the training set and testing set. ROC = receiver operating characteristic curve

Supplementary table 5. Clinical baseline data in the external validation cohort

|  | ALL  N=40 | Non- CsPCa  N=18 | CsPca  N=22 | p |
| --- | --- | --- | --- | --- |
| Age(years) | 69.3(7.99) | 64.3(6.92) | 73.5(6.32) | <0.001 |
| f/t PSA | 0.17(0.07) | 0.18(0.07) | 0.16(0.07) | 0.316 |
| Emax( kPa) | 74.3(22.6) | 66.7(21.6) | 80.5(21.9) | 0.054 |
| TZ ratio | 0.60[0.50;0.64] | 0.63[0.60;0.65] | 0.54[0.44;0.61] | 0.014 |
| Lesion density(mm/cm³) | 0.39[0.29;0.55] | 0.33[0.21;0.46] | 0.42[0.32;0.58] | 0.105 |
| PI-RADS score |  |  |  | 0.105 |
| 2 | 2(5.00%) | 2(11.1%) | 0(0.00%) |  |
| 3 | 17(42.5%) | 10(55.6%) | 7(31.8%) |  |
| 4 | 15(37.5%) | 4(22.2%) | 11(50.0%) |  |
| 5 | 6(15.0%) | 2(11.1%) | 4(18.2%) |  |
| Note: tPSA=total prostate-specific antigen; fPSA=free prostate-specific antigen; f/t PSA=free-to-total (f/t) prostate-specific antigen; Emax=the maximum value of Young's modulus in the region of interest; Emean=the minimum value of Young's modulus in the region of interest; PV= prostate volume; TZV=transition zone volume; PZV=peripheral zone volume; PI-RADS score=density prostate imaging-reporting and data system score. | | | | |


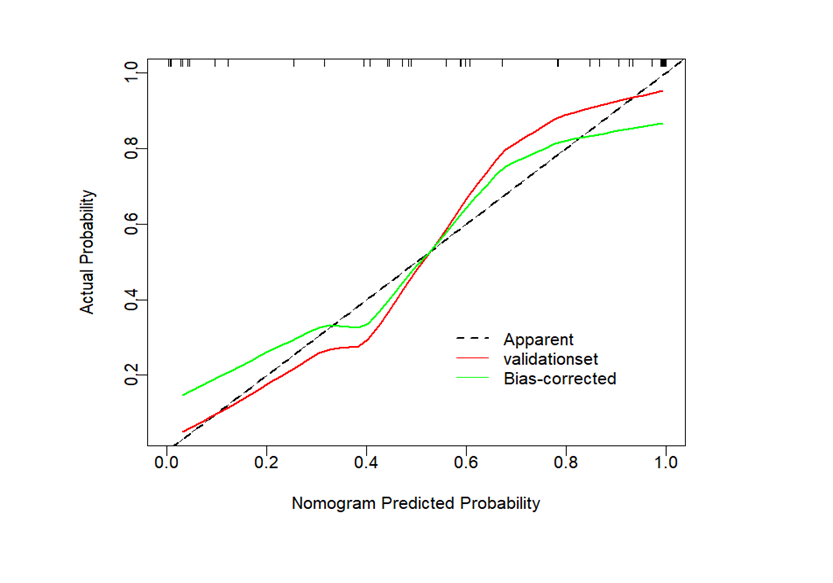


4a. Calibration curves in the external validation cohort


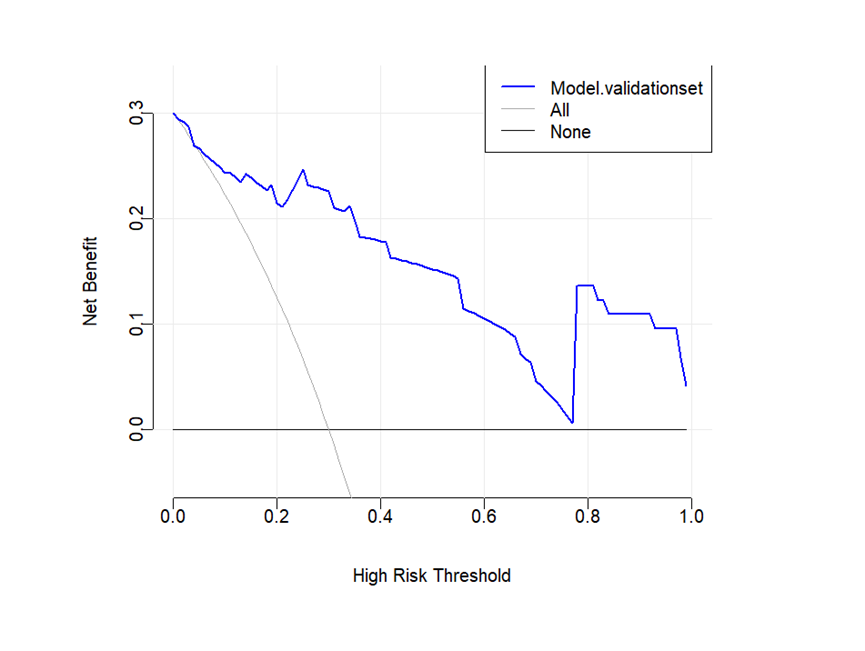


4b. The decision curve analysis in the external validation cohort


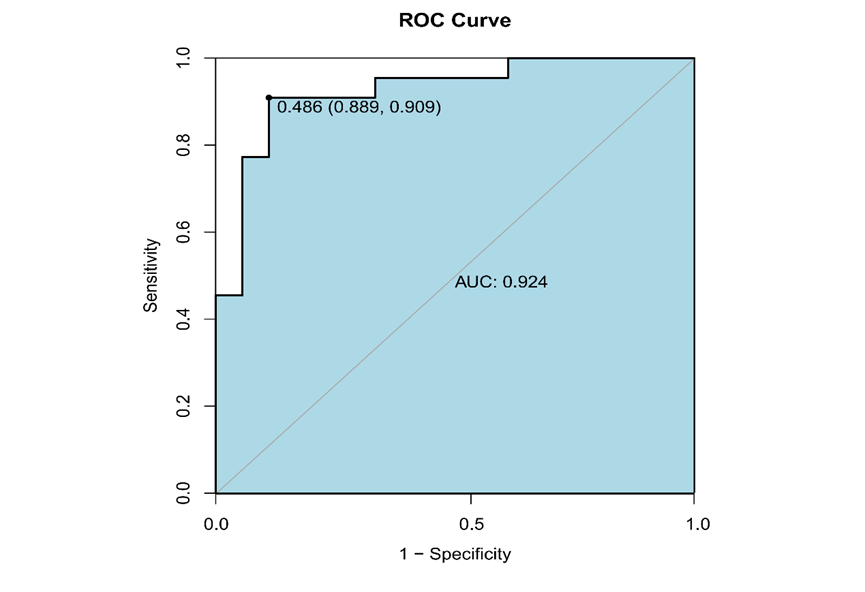


4c. ROC in the external validation cohort

Supplementary figures 4a; 4b and 4c
